# Evaluation of stability and size distribution of sunflower oil-coated micro bubbles for localized drug delivery

**DOI:** 10.1186/1475-925X-11-71

**Published:** 2012-09-20

**Authors:** Walter Duartede Araujo Filho, Fábio Kurt Schneider, Rigoberto EM Morales

**Affiliations:** 1 Department of Exact Sciences and Earth, State University of Bahia, Salvador, Bahia, Brazil; 2 Graduate Schools of Electrical Engineering and Applied Computer Science, Federal University of Technology, Curitiba, Paraná, Brazil; 3 Laboratories for Thermal Science, Federal University of Technology, Curitiba, Paraná, Brazil

## Abstract

**Background:**

Micro bubbles were initially introduced as contrast agents for ultrasound examinations as they are able to modify the signal-to-noise ratio in imaging, thus improving the assessment of clinical information on human tissue. Recent developments have demonstrated the feasibility of using these bubbles as drug carriers in localized delivery. In micro fluidics devices for generation of micro bubbles, the bubbles are formed at interface of liquid gas through a strangulation process. A device that uses these features can produce micro bubbles with small size dispersion in a single step.

**Methods:**

A T-junction micro fluidic device constructed using 3D prototyping was made for the production of mono dispersed micro bubbles. These micro bubbles use sunflower oil as a lipid layer. Stability studies for micro bubbles with diameters different generated from a liquid phase of the same viscosity were conducted to evaluate whether micro bubbles can be used as drug carriers. The biocompatibility of coating layer, the ability to withstand environmental pressure variations combined with echogenicity, are key factors that they can safely play the role of drug transporters.

**Results:**

The normal distribution curve with small dispersion of the diameter of bubbles validates the process of generating micro bubbles with low value of variation coefficient, i.e., 0.381 at 1.90%. The results also showed the feasibility of using sunflower oil as the lipid matrix with stable population of bubbles over 217 minutes for micro bubbles with an average diameter of 313.04 μm and 121 minutes for micro bubbles with an average diameter of 73.74 μm, considering bubbles with air as gaseous phase.

**Conclusion:**

The results indicate that the micro fluidic device designed can be used for producing micro bubbles with low variation coefficient using sunflower oil as a coating of micro bubbles. These carriers were stable for periods of time that are long enough for clinical applications even when regular air is used as the gas phase. Improved stability can be achieved when biocompatible gas with lower permeability is used.

## Background

Micro bubbles were initially introduced as contrast agents for ultrasound examinations as they are able to modify the signal-to-noise ratio in imaging, thus improving the assessment of clinical information on human tissue. Recent developments have demonstrated the feasibility of using these bubbles as drug carriers with an interesting feature which is appropriated for spatially limited treatment of diseases [1,2]. Well-known for its use in real-time imaging without dangerous irradiation of tissues, ultrasound can also be used now to control the drug delivery when the drug is carried by a micro bubble. This type of therapeutic application is a promising treatment modality, particularly in the cases when high concentrations of drugs are commonly administered, causing undesirable systemic side effects. The outline of these undesirable effects leads to a better quality of life of patients by reducing the possibility of secondary hospitalization during treatment.

The capability of deciding when and where in the body the micro bubbles will be fragmented resulting in localised drug delivery can potentially reduce those undesirable side effects. There are a few challenges ahead, however, and they are: (a) find compatible materials (drugs) that can be introduced into the bubbles (b) generation of bubbles with diameters smaller than 10 microns that could be introduced into the bloodstream without causing venous air embolism in the patient (c) generation of stable bubbles between the period of their generation, handling and use and (d) generation of monodispersed bubbles to increase the response to the ultrasonic field and a subsequent fragmentation.

In the dynamic process of micro fluidic-based production of micro bubbles, the bubbles are formed by a process of strangulation due to the instability at the gas/liquid interface [2-8]. Size and variation coefficient are important properties of the bubbles. The percent variation coefficient is computed dividing the standard deviation by the average size of the bubbles multiplied by 100. In microfluidic-based bubble production, the bubble size is associated to the physical properties of the liquid phase (e.g., viscosity, density), flow rates of the liquid phases, the gas pressure, the size and profile of the channels and the speed of strangulation. For a given gas pressure, there is a maximum flow rate of fluid above which the gas flow is blocked. In that case, there is no formation of micro bubbles. On the other hand, the indiscriminate increase in gas pressure can induce the atomization of the liquid phase. Therefore, the fine-tuned control of these variables in the formation of the bubbles plays a key role in the bubble size figures, and both the micro bubble producing device and the bubble liquid phase properties are essential to this technique.

Several devices have been proposed for micro bubble creation. Those include Sonication, High Shear Emulsification, Membrane Emulsification, Coaxial Electro Hydrodynamic Atomization (CEHDA) and Micro fluidic devices [2].

In this work, T-junction is used due to its simplicity and high efficiency in mono dispersed bubble production, characteristics that have been previously demonstrated [1,8]. According to Stride [8], the T-junction system can generate micro bubbles in a single step with a variation coefficient of 1%, at lower production and operational costs, since it does not require critical conditions of environmental control and cleaning, plus its ease of manufacture when compared to microlithography [9]. Furthermore, a single device can be used to produce bubbles of various sizes, based on the following control parameters: liquid flow rate, pressure, air/gas flow rate and liquid phase viscosity. In this article a micro fluidic T-junction device using 3D printing is proposed. The major advantage of this technique in relation to soft lithography [9-12] is the fabrication of devices in a single step, thus eliminating additional procedures such as the adaptation of connections and assemblies, as well as adding the potential for producing channels of circular sections, which greatly facilitates the mathematical modelling of the micro fluid dynamics associated with the formation of micro bubbles.

Micro bubbles as contrasting agents in ultrasound examinations are produced by using polymer matrices as coating layers [13-16]. Alternatively, biocompatible lipid matrices can be used. Lipid compounds represent a class consisting of one or more hydrocarbon or fluorocarbon chains covalently linked to a hydrophilic polar core, usually glycerol. Although layers surrounding the micro bubbles are less stable than the polymer, they are easier to obtain and produce more echogenic^a^ micro bubbles [17-20].

This article proposes the use of sunflower oil as lipid matrix coating, as well as studies on the dynamics of the formation of micro bubbles, the characterization of variation coefficient of the device and the stability of micro bubbles over time.

## Materials and methods

### Instrumentation

**Figure 1:**
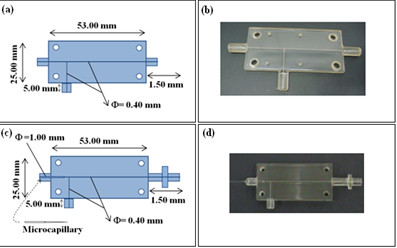
**Technical specifications of the devices used in the generation of micro bubbles. (a)** 3D printing-based prototype specifications. **(b)** Micro fluidic device manufactured using 3D printing with 0.40 *μm* channels. **(c)** Hybrid prototype specifications including 3D printing and micro capillary placement. A channel with 1 *mm* is used for positioning a micro capillary with internal diameter of approximately 20 *μm*. **(d)** Hybrid Micro fluidic device based on 3D printing and micro capillary.

Figure 1 show the devices used in the generation of micro bubbles including the technical specifications.

The T-junction devices were fabricated using a rapid prototyping machine (Objet Eden 250, Objet Geometries Ltd, Billerica, USA) with a transparent resin (FullCure720, C. Ideas, Crystal Lake, USA). This material is suitable for a wide range of rigid models, especially when the visibility of the liquid flow or internal details is necessary. To monitor the micro bubble formation a Stereo Microscope Meiji Techno model EMZ-TR (New York Microscope Company, Inc, Hicksville, USA) with a digital camera Nanosense MKIII (Dantec Dynamics GmbH, Kässboherstr, Germany) capable of a maximum frame rate of 5140 *fps* and a maximum resolution 1280 x 1024 were used.

**Figure 2:**
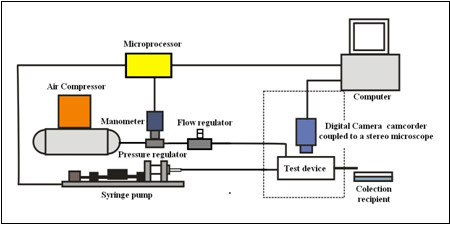
Experimental apparatus used in the generation of micro bubbles.

To control the liquid phase a syringe pump was developed to meet the demands of the flow range from 0.85 *μl/s* to 1.6 *ml/s*. The gaseous phase was supplied by an air compressor Mega Air C-6 L(Ferrari, São Paulo, BR), which can supply a maximum pressure of 792.9*kPa* at a maximum flow rate of 2.5 *l/s.* A pressure regulator Dwyer Series MPR (Dwyer Instruments, Inc, Michigan City, USA) was used to provide constant operating pressures up to 20 *kPa*. A controller SMC AS2000 (SMC Corporation of America, Noblesville, Indiana, USA) was used to allow stable operating flow rate for the hybrid-device experiments. The electronic control of the liquid flow rate and the gaseous phase (air) pressure was in charge of a Duemilanove Arduino platform (Creative Commons License Attribution- ShareAlike 2.5, Ivrea, Italy) based on the Atmel Atmega 328 microcontroller (Atmel Corporation, Colorado Springs, USA). Additionally, a computer program with a graphical user interface was developed to allow the operator to select the parameters using the computer connected to the Arduino platform through an USB serial interface. Figure 2 shows the schematic of the experimental apparatus used to generate the micro bubbles.

### Experimental procedure

**Figure 3:**
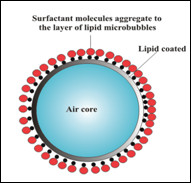
**Two-layered structure of a micro bubble.** The figure shows the two types of structures coating the micro bubble. The first is of lipid nature, and the second is formed by outermost aggregation of amphiphilic molecules to the lipid layer.

The liquid phase consisted of an emulsion composed of de-ionised water, Tween 80 and sunflower oil. Tween 80 used in the composition of the liquid phase system is a non-ionic surfactant and emulsifier derived from polyethoxylated sorbitan and oleic acid. The molecules have an amphiphilic character, which gives the ability of these molecules to accumulate at the interface of two liquids or on the surface of a liquid. Such chemical structure that has dual polarity interacts favourably with both water molecules and the molecules insoluble in water (such as vegetal oils). Due to these characteristics, the no polar part of the lipid molecules aggregate in the external layer of the micro bubble forming a bi-layer structure, as is shown in Figure 3.

The use of a surfactant increases the stability of the micro bubbles as it decreases the gas exchange with the environment external to the bubble. Phospholipid molecules could also be used to perform the same role of Tween 80 since they possess amphiphilic characteristics. According to Urdhal [21], emulsions that have low concentrations of the dispersed phase (up to 15%) can be considered as Newtonian fluids. In the specific case of the emulsions used in this study the concentrations of the dispersed phase (sunflower oil) ranged from 1% to 5%, therefore considered Newtonian fluids for viscosity calculation effects.

The concept of relative viscosity (*η*_*r*_) is used in the determination of the viscosity of an emulsion [22].

(1) $$n_{r}=\frac{n_{e}}{n_{c}}$$

Where *η*_*c*_ is the viscosity of the continuous phase and *η*_*e*_ is the viscosity of the emulsion.

The viscosities of the emulsions, which represented the liquid phase, were obtained using a rotative viscometer Brookfield DV-E (Brookfield Engineering Laboratories, Middleboro, Massachusetts, USA) at 22°C and local atmospheric pressure of 91 *KPa*.

### Bubble size distribution

For the bubble size distribution evaluation an emulsion was prepared using de-ionised water, sunflower oil and surfactant Tween 80 in the mass proportion of 100:1:2, respectively, resulting in a relative viscosity of the liquid phase, *η*_*r*_ equal to 2.24 *mPa.s*. In the 3D printing-based device we used a liquid flow rate (*Q*_*L*_) of 12 *μl/s* and an air flow rate (*Q*_*a*_) of 216.84  *nl/s*. During the bubble formation, images were captured over a period of time of 4 *s* and at a frame rate of 500 *fps*. The same procedure was carried out with the improved hybrid device (i.e., with micro capillary), using a liquid flow rate (*Q*_*L*_) 1.6 *μl/s* and air flow rate of (*Q*_*a*_) 2.73  *nl/s*.

### Bubble size determination

An image processing tool was developed in the Laboratory of Thermal Sciences (LACIT-UTFPR) to automate measurements of bubble diameter from the high frame rate video using MATLAB (MathWorks, Massachusetts, U.S.A). To determine its diameter, the bubble is segmented with the use of subtractive techniques (i.e., a reference image without bubbles is subtracted from an image under analysis). The segmented image presents the bubble region with white pixels in dark background. The number of pixels is automatically counted for each closed region so as to define the area by means of pixel counting. Using a stage micrometer the number of pixels which correspond to 100 μm is determined. This ratio (i.e., 7.45 pixels/μm) is used to define the bubble diameter using the relationship between area and diameter. From the diameter analysis statistical distribution plots can be constructed. Additionally, the variation coefficient given by the ratio between the standard deviation and average size value of micro bubbles is used.

### Bubble stability

Samples of micro-bubbles generated from each of the devices were collected on a flat glass plate containing deionised water. Images of the samples were obtained at a time interval of one minute to study the stability of the population of micro bubbles in relation to time. This study was conducted at a temperature of 22°C to a local atmospheric pressure of 91 *kPa*.

The stability of the generated micro bubbles over time was estimated from the collected samples, with the theoretical Eptein-Plesset [23,24], equation (2).

(2) $$\frac{dr}{dt}=\frac{K\left(C_{i}-C_{s}\right)BT}{M\left(P_{o}{+}^{4y}{/}_{3R_{o}}\right)}\;\left[\frac{1}{R_{o}}+\frac{1}{\sqrt{\pi \kappa t}}\right]$$

The first term of the equation, *dr/dt,* represents the rate of change of the radius of the bubble in relation to time, *k* is the coefficient of diffusivity, *B* is the universal ideal gas constant, *T* is the absolute temperature, *M* is the molecular weight of gas, *t* is time, *c*_*i*_ is the initial concentration of foreign gas in the liquid, *c*_*s*_ is the concentration of gas dissolved in the surface of the bubble, *r* is the instantaneous bubble radius, *R*_*o*_ is the initial radius of the bubble, *p*_*o*_ is the local pressure and *γ* is the surface tension between water and oil.

The equation indicates two crucial factors acting upon the stability of micro bubbles: surface resistance to gas permeation and the surface tension between water and oil. As mentioned earlier, the surfactant serves to reduce the water–oil surface tension, increasing the viscosity of the emulsion, facilitating the formation of micro bubbles and also helping to increase their stability as described in equation (2).

## Results

**Figure 4:**
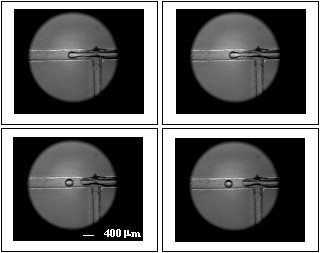
**Process of formation of micro bubbles to liquid flow rate*Q***_***L***_ **= 12** ***μl/s*and air flow rate*Q***_***a***_ **= 216.84** ***nl/s*(a) beginning of strangulation of the micro bubble.(b)** time-of pinch (separation micro bubble) **(c)** and **(d)** generated micro bubbles moving through the channel.

From the 2000 images taken, 54 different micro bubbles were detected using the initial device. Figure 4 presents the formation of micro bubbles from the onset of the strangulation, pinch-of (bubble separation) to the subsequent displacement of micro bubbles along the channel.

**Figure 5:**
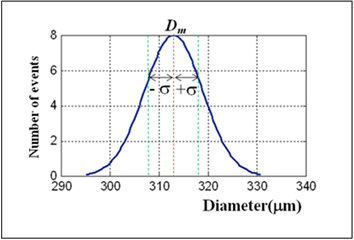
**Gaussian distribution curve of the diameter of micro bubbles as a function of number of events using the 3D printing-based device with a liquid flow rate of*Q***_***L***_ **= 12 μ*l/s*and air flow rate of*Q***_***a***_ **= 316 μ*l/s*and relative viscosity*η***_***r***_ **= 2.24** ***mPa.s*** .

**Figure 6:**
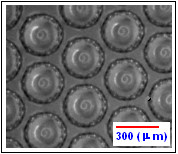
**Micro bubbles generated by the T-junction device and dispersed in aqueous media.** A central gas core and a peripheral region corresponding to lipid coating are observed in each of the structures

Figure 5 shows the Gaussian-like distribution of the diameter of micro bubbles as a function of the number of events using the initial device with a liquid flow rate of *Q*_*L*_ =12 *μl/s* and air flow rate of *Q*_*a*_ = 216.84  *nl/s* and relative viscosity *η*_*r*_ = 2.24 *mPa.s*. This result shows a high degree of uniformity of micro bubbles with diameter around the mean value (*D*_*m*_) of 313.04 *μm*, hence confirming the low degree of bubble diameter dispersion of the process with a standard deviation (*σ*) of 5.95. The uniformity of the micro bubbles can be observed in Figure 6.

The statistical analysis of the process of formation of micro bubbles to the mentioned situation points to a variation coefficient of 1.90%, thus demonstrating the device’s ability to produce micro bubbles with a high degree of uniformity in relation to the diameter. Table 1 shows the statistical data about the micro bubbles generated in the process. .

**Table 1:** Statistics of micro bubbles generated using the initial device

**Mean diameter (*D***_***m***_**) [*μm*]**	**313.04**
**Standard deviation (*σ*)**	**5.95**
**Variation coefficient (χ) [%]**	**1.90**
**Average absolute deviation** ***(δ***_***m***_**)**	**4.82**
**Perceptual Error (ε**_**p**_**)[%]**	**1.54**

Average diameter (*D*_*m*_), Standard deviation (*σ*), Variation coefficient (χ), Average absolute deviation (*δ*_*m*_) and perceptual error (ε_p_).

**Figure 7:**
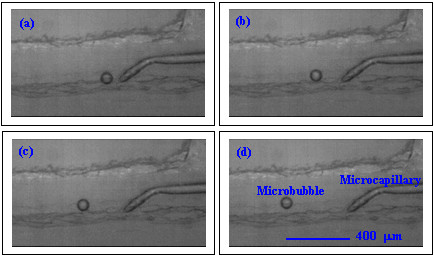
The figures (a) to (d) show a micro bubble being generated and moving along the channel highlighting the microcapillary region of the hybrid device

While the available 3D printing technology used in this work had a minimum channel diameter of a few hundred micrometers, a hybrid approach was developed to mimic a smaller channel. Therefore, a micro capillary with external diameter of 1 mm and a internal diameter of 20 μm was introduced in the air channel. From the 2000 images acquired, 52 different micro bubbles were detected using the improved device. Figure 7 shows the improved device highlighting the microcapillary region used to decrease the opening of the air channel.

**Figure 8:**
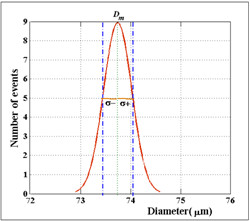
**Gaussian distribution curve of the diameter of micro bubbles as a function of number of events using the hybrid device with a liquid flow rate of*Q***_***L***_ **= 1.6*μl/s*and air flow rate of*Q***_***a***_**=2.73 *nl/s*and relative viscosity*η***_***r***_ **= 2.24** ***mPa.s*** .

Figure 8 shows the Gaussian-like distribution of the diameter of micro bubbles as a function of the number of events using the improved device with a liquid flow rate of *Q*_*L*_ = 1.6 *μl/s* and air flow rate of *Q*_*a*_ = 2.73 *nl/s* and relative viscosity *η*_*r*_ = 2.24 *mPa.s*. This result shows a high degree of uniformity of micro bubbles with diameter around the mean value (*D*_*m*_) of 73,74 *μm*, hence confirming the low degree of bubble diameter dispersion of the process with a standard deviation (*σ*) of 0.28. .

The percent variation coefficient obtained was 0.38%, thus demonstrating the device’s ability to produce micro bubbles with an even higher degree of diameter uniformity. Table 2 shows the statistical data about the micro bubbles generated in the process.

**Table 2:** Statistics of micro bubbles generated using the improved device

**Mean diameter (*D***_***m***_**) [*μm*]**	**73,74**
**Standard deviation (*σ*)**	**0,28**
**Variation coefficient (χ)[%]**	**0,38**
**Average absolute deviation** ***(δ***_***m***_**)**	**0,23**
**Perceptual Error (ε**_**p**_**)[%]**	**0,31**

Average diameter (*D*_*m*_), Standard deviation (*σ*), Variation coefficient (χ), Average absolute deviation (*δ*_*m*_) and perceptual error (ε_p_).

**Figure 9:**
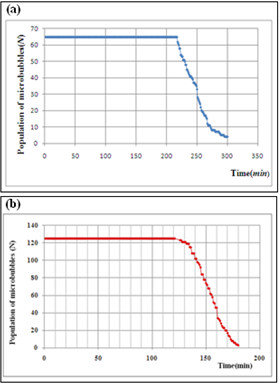
**Stability curves for the population of micro bubbles in relation to time at room temperature of 22°Cand a pressure of 91** ***KPa*and the liquid phase with a viscosity of 2.24** ***mPa.s.*(a)** Stability curve of the population of micro bubbles generated by the 3D printing-based device, to a mean diameter of 313.04 *μm*. **(b)** Stability curve of the population of micro bubbles generated by the hybrid device to a mean diameter of 73.74 *μm* .

Figure 9 shows the stability curves for each population of micro bubbles generated by two devices.

**Figure 10:**
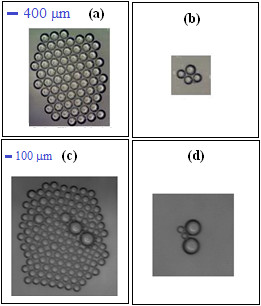
**Initial and final state of the population of micro bubbles of different diameters.** Figures **(a)** and **(b)** represent the initial and the final situation of population of micro bubbles with an average diameter of 313.04 *μm*. Figures **(c)** and **(d)** represent the initial and the final population of micro bubbles with an average diameter of 73.74 *μm* .

Figure 10 shows the initial situation and the final population of micro bubbles of different average diameter obtained from the two devices.

## Discussion

The feasibility of the use of micro bubbles as drug carrier units is closely linked to two important characteristics: stability and size uniformity of the micro bubbles. Bubble sizes with known mean value and dispersion allow the amount of carried drugs to be estimated. The stability is related to the ability of the micro bubbles to remain as individual units over time, dynamically interacting with the environment in which they are dispersed. In this type of application the micro bubbles are subjected to considerable pressure variations which can cause premature rupture of the coating layer thus causing the release of the drug outside of the chosen site. Therefore, the choice of appropriate matrices for the formation of the coating layer of micro bubbles is important as they should be biocompatible, resistant to pressure variation and echogenic. The literature shows that the most often used external layers in micro bubbles are polymeric and lipid matrices in nature.

The normal distribution curve of the diameter of micro bubbles, presented in Figures 5 and 8 as a function of event occurrence, attests the validity of micro bubble generating process when sunflower oil was used. The bubbles generated by the 3D printing-based device achieved a mean diameter of 313.04 *μm* while those produced by the hybrid device evolved to smaller bubble population with a mean diameter of 73.74 *μm*. In the 3D printing-based device the coefficient of variation was 1.9%, producing a higher value compared to that obtained by Stride [9]. This result should be associated with the control of air flow stability. Based on this hypothesis, a flow controller (i.e., SMC AS2000) was added to the experiment. The percent variation coefficient of the size of the micro bubbles obtained with the improved hybrid device was 0.38%, resulting in an improvement when compared to the 1% value found in the literature [9]. Smaller mean size and percent variation coefficient results were due to greater control of air flow associated with the coupling of the microcapillary in the air channel, whose opening diameter used was of around 20 *μm* (Figure 8). The average size of the micro bubbles generated with hybrid micro fluidic device was still seven times higher than recommended for medical applications (i.e. 10 *μm*). Finer control of air flow associated with smaller channels has clear potential for generating micro bubbles with diameter compatible for a clinical applications. Both parameters can be handled through the use of better precision flow controller and improved 3D printing technology. New additive manufacturing technology machines claim the capability of providing 16 micrometer resolution [25].

The analysis of the curves in Figure 9 showed that the choice of the lipid matrix (sunflower oil) associated to the surfactant Tween 80 responded very well with regard to stability for the mentioned mass ratios. The population of micro bubbles produced with the larger channels remained stable for 217 minutes down to a minimum value after 300 minutes. For the hybrid device, the population of micro bubbles remained constant for 121 minutes reaching the lowest value at the end of 180 minutes. This result was expected due to the smaller size of the micro bubbles. Over the time gaseous exchange occurs between the core of the micro bubble and the external environment, causing a gradual loss of gas inside the bubble to the outside environment until the total disappearance of the bubble. The more viscous the liquid phase is, the lower surface tension (*γ*) between the dispersed phase (oil) and the dispersant (water) [26] will be, leading to the formation of stable micro bubbles.

Another important factor is the formation of two-layer structures. This type of structure hinders the gas exchange between the core of the bubble and the external environment, thus enhancing its stability. In addition to the physical barrier cited, the effective diffusivity *k* of the interface associated with the molecular mass of gas (*M*) has an important role in the stability of the bubble. The constant *M* is associated with the gas used to inflate the micro bubbles. The use of gases of low water solubility and high molecular weight as the perfluorobutane (PFB) increases the survival of the micro bubbles due to their resistance to penetration in the water, combined with the difficulty of gas exchange between the gas core and the environment external to the microbubble. According to Morgan [27], PFB’s resistance to water permeation is 300 times bigger than that observed for the air. In this article the gaseous phase used in the bubbles was compressed air, whose average molecular weight is about 28.8 *g/mol*. The results suggest that the use of heavier gases with low water permeability such as the PFB whose molecular weight is 238.03 *g/mol* can increase the stability of micro bubbles compared to the results shown here. The results showed that the use of the lipid matrix coating (i.e., sunflower oil) yielded micro bubbles with potential utilisation in clinical endeavours since the bubbles proved to be stable enough outside the body. Future works may show that these bubbles can be produced with smaller sizes with the potential for collapsing with ultrasound energy, aiming at delivering drugs to specific points of the body.

## Conclusion

The results obtained in this work indicated that the stability of micro bubbles reached surprising lifetimes of over 2 hours using air as the gaseous phase. The use of higher molecular weight gases (e.g., perfluobutane) should further increase the aforementioned stability. This work has demonstrated the capability of generating micro bubbles with a very low percent variation coefficient (0.38%). This homogeneity of the population of micro bubble allow resonance with a small ultrasound frequency range allowing future control of drug delivery. Even though the generated bubble sizes were still bigger than those of clinical worth, a more refined control of air flow and improved 3D printing manufacturing can soon lead to bubbles within the range of therapeutic application.

## Endnote

^a^That responds well to the acoustic field.

## Abbreviations

*η*_*r*_: relative viscosity [*mPa.s*]; *Q*_*L*_: liquid flow rate [*ml/s*]; *Q*_*a*_: air flow rate [*ml/s*]; *fps*: frame per second; *k*: coefficient of diffusivity [*m*^*2*^*/s*]; *B*: universal ideal gas constant [*J.mol*^*-1*^*.K*^*-1*^]; *T*: absolute temperature [*K*]; *M*: molecular weight of gas [*g/mol*].; *t*: time [s]; *c*_*i*_: initial concentration of foreign gas in the liquid; *c*_*s*_: concentration of gas dissolved in the surface of the bubble [*ppm*]; *r*: the instantaneous bubble radius [*μm*]; *R*_*o*_: initial radius of the bubble [*μm*]; *p*_*o*_: local pressure [*kPa*]; *γ*: surface tension between water and oil [N/m]; χ: polydispersity index; *σ*: standard deviation [%]; *D*_*m*_: average diameter; *δ*_*m*_: average absolute deviation; €_p_: perceptual error [%]; *dr/dt*: rate of change of the radius of the bubble in relation to time.

## Competing interests

The authors declare that they have no competing interests.

## Authors’ contributions

WDAF is the principal author and his participation was instrumental in the design, development and data analysis work. FKS and REMM assisted in the research planning and data analysis, followed the progress of the work and helped with the technical reviews. All authors read and approved the final manuscript.
